# Automatic 3D Segmentation and Identification of Anomalous Aortic Origin of the Coronary Arteries Combining Multi-view 2D Convolutional Neural Networks

**DOI:** 10.1007/s10278-023-00950-6

**Published:** 2024-01-17

**Authors:** Ariel Fernando Pascaner, Antonio Rosato, Alice Fantazzini, Elena Vincenzi, Curzio Basso, Francesco Secchi, Mauro Lo Rito, Michele Conti

**Affiliations:** 1 Department of Civil Engineering and Architecture, University of Pavia, Via Adolfo Ferrata 3, 27100 Pavia, Italy; 2 3D and Computer Simulation Laboratory, IRCCS Policlinico San Donato, Piazza Edmondo Malan 2, 20097 San Donato Milanese, Italy; 3 Camelot Biomedical Systems S.r.l., Via Al Ponte Reale 2/20, 16124 Genoa, Italy; 4 Unit of Radiology, IRCCS Policlinico San Donato, Piazza Edmondo Malan 2, 20097 San Donato Milanese, Italy; 5 Department of Congenital Cardiac Surgery, IRCCS Policlinico San Donato, Piazza Edmondo Malan 2, 20097 San Donato Milanese, Italy

**Keywords:** Convolutional neural network, Coronary arteries, AAOCA, U-Net

## Abstract

This work aimed to automatically segment and classify the coronary arteries with either normal or anomalous origin from the aorta (AAOCA) using convolutional neural networks (CNNs), seeking to enhance and fasten clinician diagnosis. We implemented three single-view 2D Attention U-Nets with 3D view integration and trained them to automatically segment the aortic root and coronary arteries of 124 computed tomography angiographies (CTAs), with normal coronaries or AAOCA. Furthermore, we automatically classified the segmented geometries as normal or AAOCA using a decision tree model. For CTAs in the test set (*n* = 13), we obtained median Dice score coefficients of 0.95 and 0.84 for the aortic root and the coronary arteries, respectively. Moreover, the classification between normal and AAOCA showed excellent performance with accuracy, precision, and recall all equal to 1 in the test set. We developed a deep learning-based method to automatically segment and classify normal coronary and AAOCA. Our results represent a step towards an automatic screening and risk profiling of patients with AAOCA, based on CTA.

## Background

Anomalous aortic origin of the coronary arteries (AAOCA) is a rare congenital disease in which one of the coronary arteries may originate from the aorta but in an abnormal position. The AAOCA are highly heterogeneous and generally are classified depending on the affected coronary, the location of the ostium, the course, and the geometrical characteristics of the anomalous artery [1]. Often the diagnosis of AAOCA is incidental during examinations for other medical reasons, although in a significant proportion of the cases, the first manifestation of the disease may be a sudden cardiac death (SCD) event or a myocardial infarction under effort conditions [2, 3]. Because AAOCA, which is one of the leading causes of SCD in young athletes [4, 5], may also have a lethal presentation in the late adult age, it has been considered in differential diagnosis of myocardial infarction with non-obstructive coronary arteries [6] and its diagnosis cannot be missed because it may deeply impact the patient’s outcome.

Contrast-enhanced coronary CT angiography (CTA) has become the first-line diagnostic tool for evaluating chest pain or myocardial infarction in the adult population [7], because it accurately depicts the coronary tree and the relative stenotic lesions without any invasive intervention such as for the coronary angiography. For this reason, the number of coronary CTAs performed has dramatically increased, leading also to the increase in diagnosis for AAOCA. When an AAOCA is detected in an asymptomatic subject, the decision about which is the best treatment is greatly debated and mainly relies on anatomical characteristics, result of functional tests, and invasive evaluation [8].

Quantitative analysis of AAOCA from CTA is used in different contexts, such as diagnosis and risk profiling, surgical planning, and numerical biomechanical simulations [9–12]. Recently, deep learning techniques have shown promising performance in the medical field, addressing classification, segmentation, and detection tasks [13–15]. Since segmentation is a time-consuming and subjective task, an automatic tool capable of extracting the coronary lumen from the CTA would reduce the inter- and intra-operator variability and would enable a faster analysis of large datasets. Moreover, coronary arteries segmentation is a useful step in performing coronary examination and accurately identifying vessels with anomalous origin and course [16].

The potential of deep learning and convolutional neural networks (CNNs) for coronary artery segmentation in CTA images has been demonstrated in several studies [17]. Different variations of CNNs have been explored, including 3D CNNs, multi-channel U-Nets, and graph CNNs, with reported Dice score coefficients (DSCs) ranging from 0.6 to 0.9 [18]. Recent review works have highlighted the growing popularity of CNN-based methods for coronary artery segmentation and emphasized the need for further development to translate research into clinical practice [17, 19].

Therefore, we aimed to create an AI-based automatic tool, by developing a CNN, able to accurately segment the coronary tree in AAOCA patients and automatically detect the presence of the anomaly based on the segmented geometry. We have already conducted some preliminary experiments in which we developed a CNN to automatically segment the aorta from CTA images, obtaining a mean DSC of 0.93 [20]. Following these encouraging results, in the present work, we addressed the feasibility of fully automatic coronary segmentation and classification.

## Methods

### Image Acquisition and Preprocessing

We included 124 CTA scans of patients (age: 36 ± 20 years old, 65% male) referred to IRCCS Policlinico San Donato (Milan, Italy), with either normal (*n* = 50) or anomalous aortic origin of the coronary artery (*n* = 74). For each CTA scan, the aortic root and the coronary arteries were semi-automatically segmented by a trained expert (AR) using ITK-Snap interactive tool [21], and considered as the ground truth. The study was approved by the Ethical Committee (protocol number: 54/INT/2023), and the consent was waived due to the retrospective nature of the study.

Image pre-processing plays a crucial role in CNNs training [22]; therefore, after manual segmentations were conducted and prior to CNN training, all CTA scans underwent the following steps. First, volumes were resized to a fixed dimension of 320 × 320 × 320 voxels to account for heterogeneity among scans. Then, we set a Hounsfield units (HU) window of interest centered at 100 HU and width 700 HU, i.e., between − 250 HU and 450 HU. All voxels with HU outside of this range were saturated.

### Workflow Description

We implemented three identical bidimensional Attention U-Nets [23] for each of the CTA orthogonal planes (axial, sagittal, and coronal), which were reconstructed from the preprocessed CTA volume. Each single-plane network performed multi-class classification of the input pixels onto the three considered classes, namely, background, aortic root, and coronary arteries. The output of each CNN consisted of three 2D probability maps (one for each class) in which the intensity value of the pixels represented the probability of belonging to each class. Then, given the single-view probability maps obtained for the entire CTA, a simple average was performed to integrate the results among the three orthogonal directions.

**Fig. 1:**
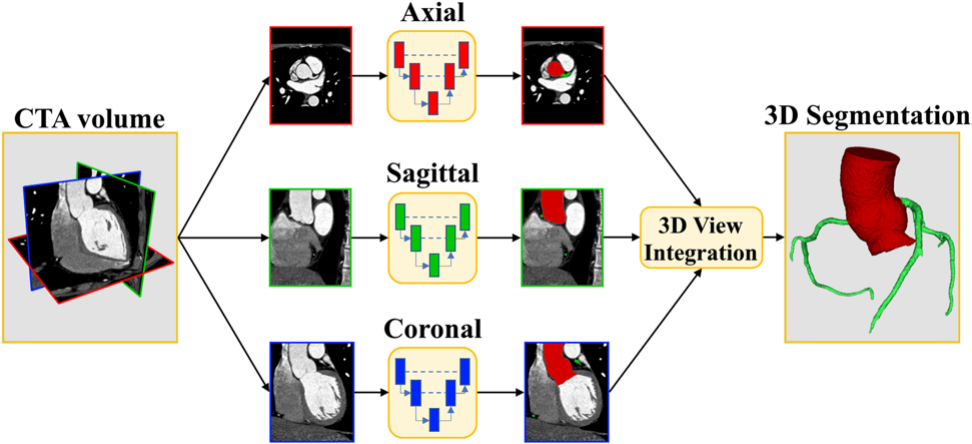
Schematic of the developed workflow. Three 2D U-Nets were implemented, corresponding to the axial, sagittal, and coronal planes. The networks’ outputs were integrated into a single 3D label map

For each class of interest (i.e., aortic root, and coronary artery), a 3D label map was obtained by binarizing the integrated prediction maps with the threshold that maximized the DSC on the validation set for that specific class. The voxels in which the probability of more than one class surpassed the binarizing threshold were assigned to the class with highest probability. The end-result was a single 3D label map (i.e., the output of the multi-class classification task) representing the segmentation of the aortic root and the coronary arteries within the CTA volume. Figure 1 shows a schematic representation of the workflow.

The 3D view integration step was intended to regularize the voxel prediction by considering the spatial information from the three orthogonal views. In other words, the predictions made by the three 2D U-Nets were combined to provide a spatially coherent final segmentation, overcoming the limitations of single-plane CNNs, namely the lack of information regarding the association among different slices.

The CNNs were implemented in Python, using Keras framework (v 2.8.0) based on Tensorflow (v 2.8.3) with GPU support (NVIDIA GeForce RTX 2080 Ti graphic card with CUDA compute capability of 7.5), under Windows 10 operating system.

### CNN Training

The CTA acquisitions and the corresponding segmentations were randomly divided into three groups: training set (*n* = 99 scans), validation set (*n* = 12 scans), and test set (*n* = 13 scans). Each CTA scan was parsed into 2D axial, sagittal, and coronal views, and the single-plane CNNs were trained for each orthogonal view using a separate segmentation model. To increase the variety of the dataset, we performed data augmentation on the training and validation by making a random rotation (range: − 7°–7°), shift (range in width direction: − 22–22 pixels; range in height direction: − 22–22 pixels for axial planes and − 72–72 pixels for sagittal and coronal planes) and zoom (range: 0.85–1.15) to each image.

To train the models, we used binary cross-entropy as a loss function, and Adam optimizer with learning rate = 0.0001 to optimize the network parameters. We trained the CNNs providing to the 2D networks mini-batches of 10 slices each, randomly sampled from the training set. The training was stopped using early stopping criteria, with patience of 15 epochs.

### Classification of Anomalous Coronaries

The classification task was divided into two parts. First, a connectivity criterion of the 3D segmentation was applied. A correct segmentation of normal coronaries should consist of two connected components, one for each trunk (left and right). In some anomalous cases, both coronaries share the same ostium, resulting in a single connected component on the segmentation. Furthermore, in another type of anomaly, the left anterior descending and the left circumflex arteries do not originate from the left main coronary, but instead both originate directly from the aortic root. In this case, the segmentation results in three connected components. Accordingly, the first step was to classify a CTA as anomalous if its segmentation possessed either one or three 3D connected components. In cases with a segmentation composed of exactly two 3D connected components, an origin angulation criterium was applied, as explained in the following paragraph.

**Fig. 2:**
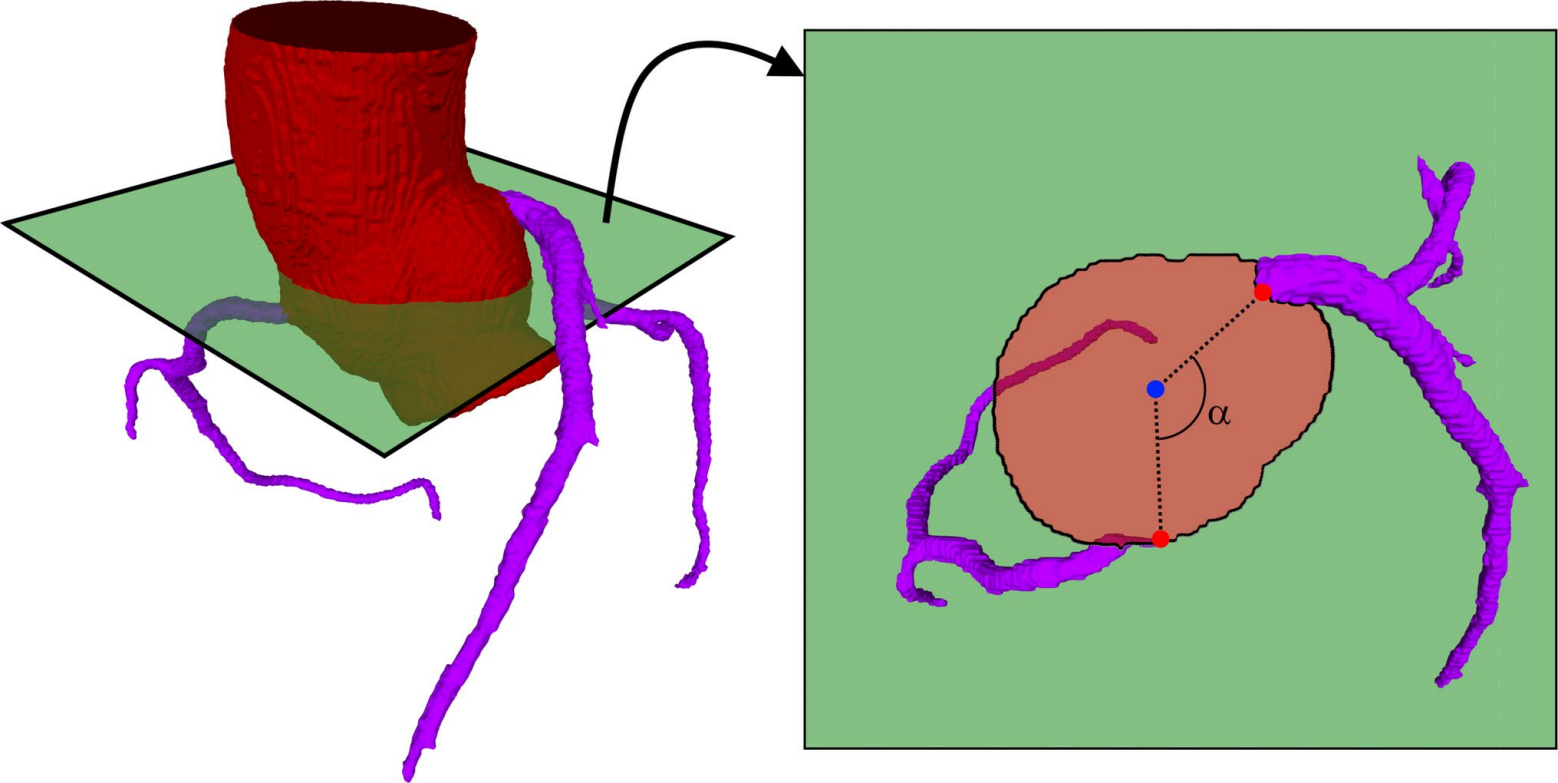
Example of the calculation of the origin angle. On the left is shown the segmentation of a normal case from the test set, in which the ostia of the coronaries were automatically retrieved. The green plane is the axial slice that is halfway between the Z coordinates of the origins. The points where the ostia were found were projected onto this plane and the centroid of the aorta was automatically computed (right side of the figure). The origin angle (α) was computed between the vectors joining the centroid (blue point) with the ostia (red points)

The ostia of the anomalous coronary arteries are close to each other, whereas in the case of normal coronaries the ostia are far apart. Using this anatomical characteristic, to classify the coronaries as normal or anomalous, we first identified the coronary ostia as the points within the segmentation that were closer to the aortic wall. Then, the 3D coordinates of the origins were projected onto the axial plane that was halfway between the Z values of both origins; and in that plane the centroid of the aorta was automatically obtained. Finally, we calculated the directions between: (i) the aortic centroid and the first coronary ostium, and (ii) the aortic centroid and the second coronary ostium (Fig. 2). The angle between these directions (origin angle, α) was used to discriminate between normal and abnormal coronaries using a decision tree model.

### Data Analysis

The segmentations obtained with the developed method were compared against the ground truth annotations using the DSC, which is commonly used to evaluate the performance of the multi-view network as an overlap measure between the predicted and the ground truth segmentations. Two different DSC were computed, i.e., one DSC for the aortic root segmentation and one DSC for the coronary segmentation. The classification results were evaluated using accuracy, precision, and recall.

## Results

The output of each CNN consisted of 2D probability maps for the aortic root and the coronary arteries, which were binarized with the thresholds that maximized the DSC on the validation set. The resulting thresholds were 0.30 and 0.15, respectively.

**Fig. 3:**
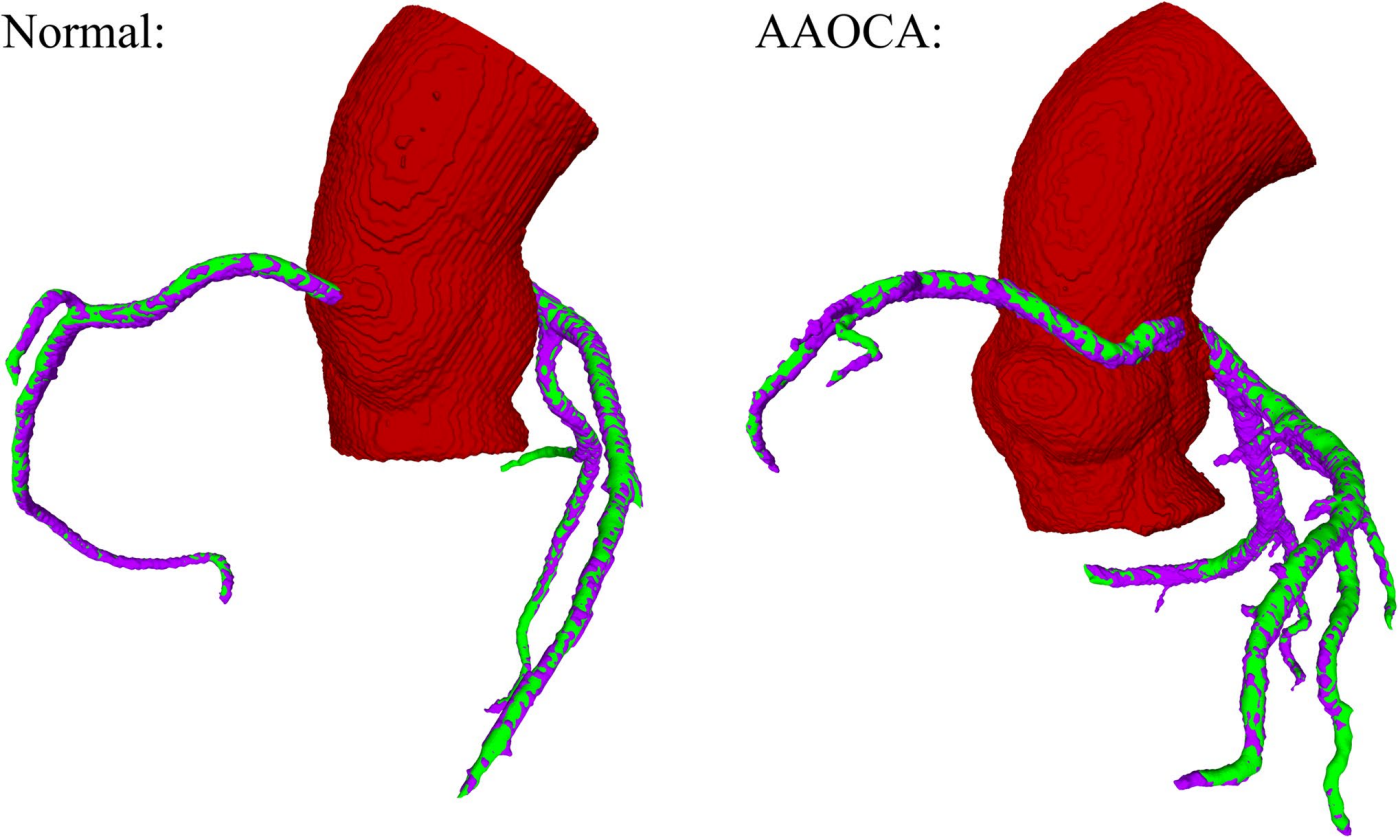
Examples of segmented aortic root and coronary arteries from the test set. On red is shown the segmentation of the aortic root, and on green and purple, the ground truth and the segmented coronary arteries, respectively. It can be observed a good correspondence between the coronary arteries segmented with our proposed method and the ground truth (superposition of green and purple). On the left, a normal case is shown, where both coronaries originate from the corresponding physiological site; and on the right, an AAOCA case is shown, where it can be seen the proximity between the ostia of the coronaries

Figure 3 shows two examples of the overlap between the ground truth and the automatically obtained segmentations of a normal and an AAOCA case. Both cases shown belong to the test set. It can be observed that there is a high superposition between both segmentations.

Table 1 shows the mean DSCs obtained for the aortic root and the coronary arteries in all sets of CTAs, namely the training, validation, and test sets. It can be observed that, on the test set, the DSC for the aortic root was 0.95 ± 0.04, whereas the DSC of the coronary arteries was 0.80 ± 0.11. Possible reasons for this difference are addressed in the “Discussion” section.

**Table 1:** Results of segmentation (DSCs for the aortic root and for the coronary arteries) and classification (accuracy, precision, and recall) for training, validation, and test sets

		**Training set (*n* = 99)**	**Validation set (*n* = 12)**	**Test set (*n* = 13)**
**Segmentation**	DSC aortic root	0.98 ± 0.03	0.97 ± 0.02	0.95 ± 0.04
	DSC coronary arteries	0.84 ± 0.06	0.81 ± 0.09	0.80 ± 0.11
**Classification**	Accuracy	0.99	1	1
	Precision	0.98	1	1
	Recall	1	1	1

Table 1 shows the accuracy, precision, and recall of the classification task. Normal and anomalous CTAs showed a median of the origin angle of 135° (quartiles: 121°–154°) and 30° (quartiles: 20°–46°), respectively, with a threshold of 73°. In almost all cases, the decision tree correctly classified the coronary arteries as normal or anomalous. There was only one CTA incorrectly classified as abnormal. In no cases, there was an anomalous coronary tree wrongly identified as normal.

**Fig. 4:**
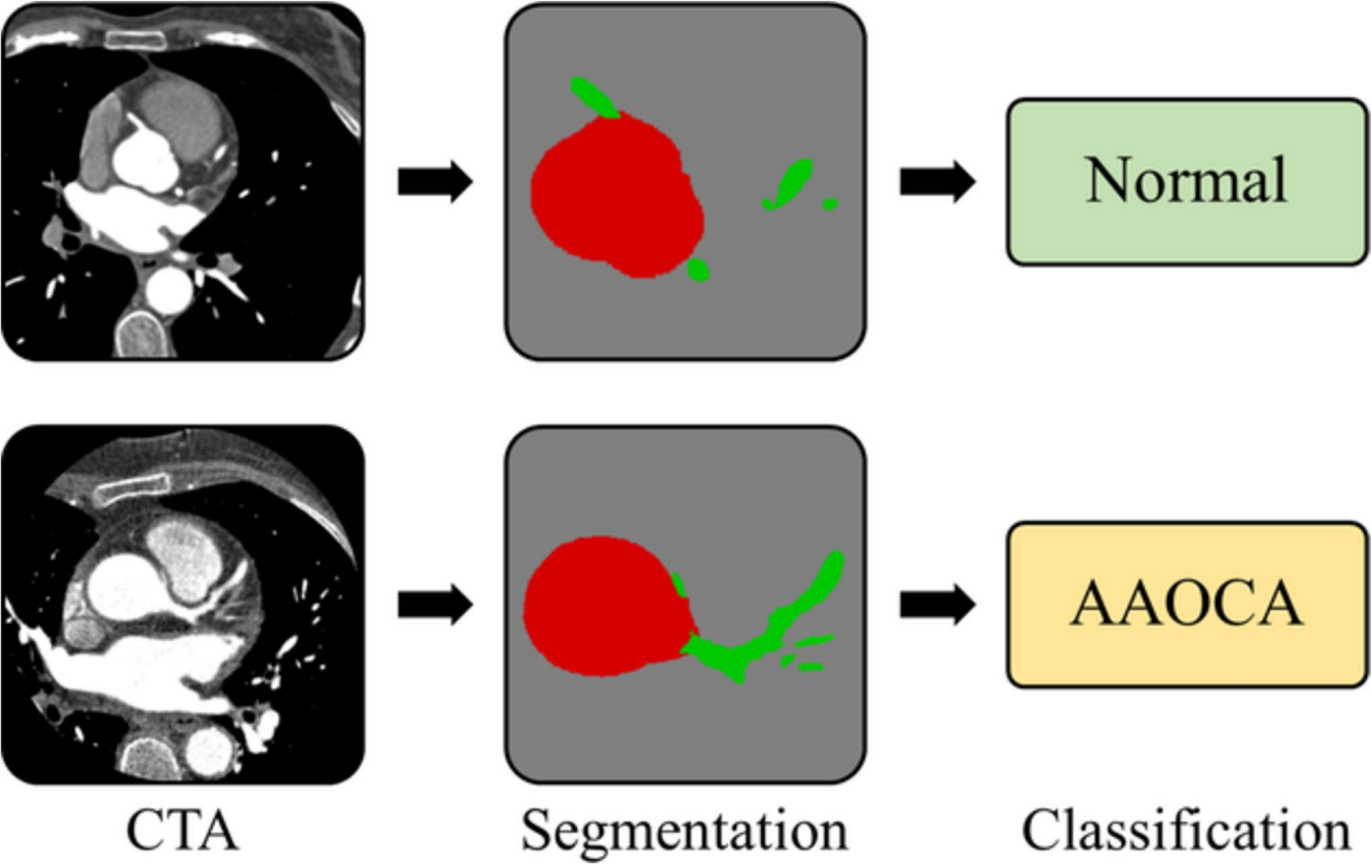
Examples of axial input images (left) with the corresponding segmentation outputs (center) and classification labels (right). Colors gray, red, and green on the segmentation images correspond, respectively, to background, aorta, and coronary arteries. It is noteworthy that the segmentation images are not to scale with respect to the CTA images to allow better visualization of the area of interest; all unshown pixels were assigned to the background. The examples correspond to a normal (top row) and an AAOCA (bottom row) patient from the test set. In both cases, the assigned label matched the true class of the patient

Figure 4 shows two examples of input images from the test set (one normal and one AAOCA) with the respective output images (segmentation) and classification labels.

## Discussion

We developed a fully automatic method for segmenting the aortic root and either normal or anomalous coronary arteries from CTA images based on 2D attention U-Nets with multi-view integration. Moreover, the segmented geometries were automatically classified as normal or anomalous using 3D connectivity features and a decision tree model.

Segmentation of the aortic root showed high performance, with a mean DSC of 0.95 in the test set. The coronary tree segmentation showed a mean DSC of 0.80 on the test set. This lower performance can possibly be explained by a particular anomaly that was included in the dataset, i.e., the retroaortic origin [1], which is a rare type of AAOCA. In this case, the anatomy of the coronary arteries differs substantially from other types of AAOCA. Our dataset possessed 2/99, 2/12, and 3/13 cases of retroaortic anomalies in the training, validation, and test sets, respectively. Given the low proportion of these cases in the training set, the CNN did not learn to generalize this type of abnormality. The DSCs of retroaortic cases were in the range of 0.5–0.7, significantly lowering the mean value of the entire sets (including the training and validation sets). Accordingly, the median DSCs of the training, validation and test sets were 0.87, 0.83, and 0.84, respectively, which is higher than the mean value in all cases. Moreover, the mean DSC on the training set, considering only the 10 non-retroaortic cases, was 0.83. Future works should include larger datasets with significant amounts of CTAs of all the types of AAOCA to be able to generalize this highly heterogeneous anatomy.

The classification task provided excellent results, showing perfect predictions in the validation and test sets. The single case that was misclassified corresponded to a normal coronary tree that was considered as anomalous. We highlight that in none of the analyzed CTAs an anomalous case was missed and classified as normal. From the clinical point of view, this a major finding, since missing pathological cases would imply an under-diagnosis, leading to potentially severe complications. Instead, the wrongly classified as anomalous case would be immediately identified as normal by physicians, given the geometry of the segmentation. The misclassification was caused by a small group of voxels near the aortic root that was considered as part of the right coronary artery in the automatic segmentation. Since these voxels were not 3D connected to the rest of the right coronary artery, the segmentation resulted in three connected components, which were thus automatically classified as anomalous (without considering the angle of origin). To avoid this issue, the classification method could be improved by adding a size criterion on the connected components; the volume of the region wrongly identified as artery was 15 ml, representing 0.68% of the total volume of the segmented coronary tree. A threshold could be applied to eliminate small regions like these from the automatic segmentation before performing the classification task.

Deep learning is now the most used tool to segment cardiac images, although a small number of works investigated its use for the segmentation of coronary arteries [13]. Some authors have focused on the extraction of the centerline [24, 25] or of the centerline and the radius [26]. Although in many cases the geometry of the vessel may be assumed cylindrical, rending sufficient the information of centerline and radius, patients with AAOCA often present an intramural tract, which has a highly elliptic cross section [9], thus needing a volumetric segmentation. Other authors have used CNNs to achieve an end-to-end 3D segmentation of the coronary arteries [27–29], obtaining DSC that ranged between 0.6 and 0.8. Some authors have included anatomical restrictions to regularize during training [30, 31]. However, in the context of pathological anatomies like the ones considered in our work, these types of constraints are not suitable to be applied [13] (particularly in a rare disease like AAOCA, in which there exists a large variety of possible anatomical configurations). Recent works have implemented different variations of U-Nets to segment the coronary arteries, being one of the most utilized architectures for this purpose. Using a modified 2D U-Net architecture, Cheung et al. obtained a DSC of 0.888 in the segmentation of the coronaries of intermediate risk patients with anatomically normal arteries [18]. To our knowledge, ours is the first work to assess the feasibility of 3D segmentation using U-Nets in patients with AAOCA. Furthermore, compared to the available literature, we obtained high DSC values for the coronary arteries alone, as well as for the aorta (above 0.80 and 0.95, respectively).

The performance of 2D compared to 3D CNNs has been discussed [18]. 2D approaches are easier to implement and usually converge faster than 3D CNNs, which, in turn, allow a volumetric interpretation of the input data instead of analyzing single planes. Nevertheless, the combination of both features (easy and fast implementation from 2D and volumetric information from 3D) is possible thanks to the so-called 2D + approaches. One example of a 2D + workflow is the one adopted in this work; namely, the multi-view integration, which uses a single 2D CNN for each orthogonal plane, thus enabling a 3D coherent end-result since information among slices is taken into consideration.

Although the current dataset has some limitations in terms of available CTAs, the segmentation model showed promising results. In future developments, having more CTA scans, it will be possible to perform a more accurate and comprehensive comparison between our pipeline and state of the art methods for segmenting coronary arteries in AAOCA.

A limitation of our work was the inclusion of several different types of AAOCA anatomies into the dataset. Better results might be obtained if a single type of anomaly was considered or if a separate deep learning system was developed for each sub-classification of AAOCA disease. However, any of these options would require a larger dataset, containing a wide range of AAOCA variants. Given the rareness of AAOCA disease and the substantial number of undiagnosed cases, data collection is a slow process. Future works should include larger datasets.

In this work, we trained a deep learning model with a single configuration of training, validation, and test sets. It could be helpful to perform cross validation (e.g., five-fold cross-validation) to have an average performance evaluation on training, validation, and test set. The reported results might then be more stable, also reducing the impact of outliers in performance evaluation. By training and testing on different subsets of data, a more complete understanding of the performance of the model on different CTAs will be obtained, both for the segmentation and the classification tasks. This can be very important when working with limited datasets, as choosing a single configuration for training, validation and testing sets can lead to bias.

## Conclusion

We developed a deep learning-based workflow to automatically segment the aortic root and both normal and AOOCA coronary arteries using single 2D U-Nets with multi-view integration. Furthermore, the segmented geometries were automatically classified as normal or anomalous using 3D connectivity and a decision tree model. We obtained high correspondence of our segmentation with respect to the ground truth and excellent results in the classification task. Further work is needed to develop tools that could be used in clinical routine.

## Data Availability

The data that support the findings of this study are available from the corresponding author upon reasonable request.
